# Economic complexity of cities and its role for resilience

**DOI:** 10.1371/journal.pone.0269797

**Published:** 2022-08-04

**Authors:** Athanasios Lapatinas, Anastasia Litina, Konstantinos Poulios

**Affiliations:** 1 European Commission, Joint Research Centre (JRC), Ispra, Italy; 2 University of Macedonia, Thessaloniki, Greece; 3 University of Ioannina, Ioannina, Greece; Tohoku University, JAPAN

## Abstract

The aim of the paper is to propose the construction of an index that captures the economic complexity of cities over the globe, as well as to explore whether it is a good predictor for a range of city-level economic outcomes. This index aspires to mitigate data scarcity for cities and to provide policy makers with the tools for monitoring the evolving role of cities in the global economy. Analytically, we implement the economic complexity methodology on data for the ownership, location and economic activities of the world’s 3,000 largest firms and their subsidiaries to propose a new indicator that quantifies the network of the largest cities worldwide and the economic activities of their globalized firms. We first show that complex cities are the highly diversified cities that host non-ubiquitous economic activities of firms with global presence. Then, in a sample of EU cities, we show that complex cities tend to be more prosperous, have higher population, and are associated with more jobs, human capital, innovation, technology and transport infrastructure. Last, using OLS methodology and accounting for several other confounders, we show that a higher ECI, at the city level, enhances the resilience of cities to negative economic shocks, i.e., their ability to bounce back after a shock. Specifically, we find that the expected increase of the ratio of employment in 2012 over 2006 is 0.01 (mean: 0.992; standard deviation: 0.081) when the ECI increases by 1 unit (mean: 0.371; standard deviation: 1.094), i.e., a satisfactory pace of recovery, in terms of employment. The ability to diversify in the presence of a shock, the reallocation of factors of production to other sectors and the ability to extract rents associated with those diversified activities, uncovers the mechanics of the ECI index.

## Introduction

The multinational firms and their main vehicle, foreign direct investment (FDI) are key drivers of the global economy. Cities all over the globe compete to attract and maintain globalized firms with stable or rising market shares in their economic sector activity [1]. Firms with global presence are associated with positive attributes for the urban economies and the desirability of FDI is backed by an extensive literature (see e.g. [2–5]).

The capability of a city to attract globalized firms and investors, which undoubtedly contributes significantly to its long-run development and resilience, depends on a multitude of factors ranging from human capital and skills to particular resources and institutions [6, 7] (By the term resilience we refer to the ability of a region to bounce back after a shock as defined in [52]). Depending on the availability of such factors some cities are more competitive in attracting high-tech industries and knowledge-intensive services. Developing urban competitiveness based on specialization in certain economic activities or choosing a more diversified economic structure is a crucial urban planning decision. On the one hand, the new growth theory literature concludes that specialization leads to higher productivity growth in the form of learning [8–10] and according to the ‘Ricardian’ view, specialization matters because some economic activities provide larger growth opportunities than others [11]. On the other hand, specialization in a single economic activity or a restricted set of activities, can be harmful for cities because of the low adaptability and higher vulnerability to external economic shocks, especially shocks that may affect this particular sector [12]. If a specific economic activity is severely hit by a shock then the cities that specialize in this activity will be more affected and will experience a deeper recession compared to the ones with a more diversified economic structure [13].

For policy makers, understanding the global urban landscape and how economic activities are linked to geographic locations is valuable for monitoring the urban trends and anticipate challenges that emerge from cities’ management. Our study quantifies the economic dependencies between cities worldwide and links them to the diversification and ubiquity of economic activities of their globalized firms. Due to data scarcity for cities, policy makers have to deal with a shortage of available tools for monitoring the evolving role of cities in the global economy and their competitive characteristics that attract foreign capital, firms and people [14, 15]. We propose a new index, with the aim to help policy makers that seek to improve their evidence-based decisions with new monitoring and evaluation tools and to support companies in tracking the evolving role of cities worldwide and positioning their business and investment strategies accordingly. Our index offers insights on the choices faced by companies that are looking for new markets and by policy makers who seek to improve their urban management and the alignment of their diplomatic efforts with their cities’ FDI interests [16].

In this study, by combining concepts of the economic complexity methodology, economic geography and urban planning we compute the economic complexity index (ECI) for 1, 169 cities around the world. The index measures the composition of a city’s “pool” of economic activities by combining information on the diversity of economic activities (the number of different economic activities in the city) and the ubiquity of economic activities (the number of other cities with these economic activities). The intuition is that relatively high scores on the ECI indicate cities that are diverse and have economic activities that, on average, have low ubiquity, i.e., they are placed in only a few other cities.

The ECI for cities is an indirect measure of cities’ competitiveness. Using the Hidalgo’s metaphor of “bucket of Legos” [18], the economic activities of globalized firms are equivalent to a Lego block and a city is equivalent to a bucket of Legos. Cities will be able to attract globalized firms (economic activities) for which they have all the necessary competitive/determinant factors such as labour and human capital (population and ‘person-bytes’ [17]), infrastructural capital (transport accessibility and performance and digital infrastructure), available knowledge and technology (innovation capacity), institutions (e.g. level of development), just like a child is able to construct a Lego model if the Lego-bucket contains all of the necessary Lego blocks. Following Hidalgo and Hausmann’s analogy, we argue that the ECI for cities is a tool for inferring the various determinants of urban competitiveness for economic gain, i.e. the Lego pieces inside a child’s bucket, by looking only at the models (connections between cities and economic activities of their globalized firms) that children (i.e., cities), each with a different bucket of Legos can make. In other words, a high score in the ECI, i.e., if the city hosts globalized firms that operate in complex economic activities (i.e. highly diversified and non-ubiquitous economic activities), signals the availability of the above determinants of competitiveness in a city.

The economic complexity methodology was originally established with main vehicle the bipartite network between countries and exported products [18]. Trade data are commonly used because their classifications are internationally comparable. However, the application of the methodology to other datasets has been accelerating in recent years and includes data on technological innovation (patents), research papers, industries, green products, occupations and diseases [19–25] (In a recent paper, Hidalgo [26] provides a thorough review of economic complexity theory and its applications).

To the best of our knowledge this is the first work that applies the economic complexity methodology on data about location, ownership and economic activities of the world’s largest firms and their subsidiaries, and analyzes the bipartite network of cities linked to the economic activities of their globalized firms. Analyzing the structure of this bipartite network we find similarities with the bipartite network of international trade data. Specifically, we show that the *city-activities of globalized firms network* is organized in a nested pattern which implies that a city’s diversity tends to correlate negatively with the average ubiquity of the economic activities of its globalized firms [26, 27].

The ECI for cities measures whether a city hosts economic activities that are located in the densely connected core of the economic activities space i.e., whether many other economic activities of firms with global presence occur in many other cities. The nestedness property of the network signals that the complex economic activities (i.e., the nodes in the dense core of the network) agglomerate relatively more and concentrate in only few diverse cities. Furthermore, similar nested structures found in ecology (mutualistic networks) where species (nodes) interact in a mutual beneficial way can be seen as evidence that complex cities are relatively more economic stable and compatible with globalized firms’ coexistence [28–32]. Cities and globalized firms when seen as a mutualistic ecosystem also exhibit higher resilience against economic shocks [33–37].

To this end, our work also contributes to the rapidly growing literature on cities’ (regional) economic resilience by analyzing the relationship between resilience and the proposed economic complexity metric for cities. To the best of our knowledge this approach is novel to the relevant literature (see e.g. [38–51]). Some recent works consider the role of knowledge and technology networks’ structure on regional resilience [52–54].

Based on the above, the aim and contribution of this paper is twofold: (*i*) to illustrate how employing the economic complexity methodology allows us to develop a new tool for policy makers. This tool quantifies the economic activities space at the city level and could support the planning policies and strategies for cities; (*ii*) to highlight the value added of the proposed tool by illustrating how a city’s economic complexity is associated with various socio-economic indicators and its resilience to economic shocks.

The remainder of the paper is structured as follows. Section The city-activities of globalized firms network describes the *city-activities of the globalized firms network* which forms the analytical backbone of our study. Section The economic complexity of cities as derived from the economic activities of their globalized firms presents the application of the economic complexity framework in developing the ECI for 1, 169 cities worldwide using data for the ownership, location and economic activities of the world’s 3, 000 largest firms and their almost one million subsidiaries. Section The economic complexity of cities across the world shows the results of the structural analysis of the city-activities of globalized firms network, with a particular focus on cities both worldwide and in Europe. Section Linking economic complexity to socio-economic indicators of cities empirically investigates the association of the economic complexity of cities with various socio-economic indicators at the EU regional level. In particular, we employ a sample of EU cities-regions for which several consistent economic measures are available. Our unconditional correlations suggest that: (*i*) complex economic activities of globalized firms concentrate in large and prosperous cities (subsection Complex economic activities of globalized firms concentrate in large and prosperous cities); (*ii*) complex economic activities of globalized firms concentrate in cities with increasing concentration of jobs, human capital and innovation (subsection Complex economic activities of globalized firms concentrate in cities with increasing concentration of jobs, human capital and innovation); (*iii*) complex economic activities of globalized firms concentrate in cities with robust digital and transport infrastructures (subsection Complex economic activities of globalized firms concentrate in cities with robust technology and transport infrastructure). Section Complex economic activities of globalized firms concentrate in cities with robust technology and transport infrastructure discusses the econometric specification adopted for the analysis of the relationship between economic resilience and cities’ economic complexity and presents the results of the estimations. Finally, in section Conclusions, we conclude offering some remarks on how the proposed ECI at the city level could be considered in policy-decision making on improving urban competitiveness in attracting FDI, knowledge/information and people. We also discuss the limitations of our study and suggestions for future research.

## The city-activities of globalized firms network

### Data on firms with global presence

Information on firms/sectors comes from the BvD *Orbis* database for the year 2010. Following [13] we use data for the 3, 000 largest firms with global presence and their almost 1 *million* direct and indirect links to almost 800, 000 subsidiaries and we aggregate firm locations using the concept of *Functional Urban Areas* (FUA) which agglomerates municipalities according to their functional orientation in a consistent way across countries. This methodology has been jointly developed by the OECD and the European Commission (*European Spatial Planning Organization Network*—ESPON) using population density and travel-to-work flows as key information. Hence, a FUA consists of a densely inhabited city and of a surrounding area (commuting zone) whose labour market is highly integrated with the city [55]. More details on how firms are classified and what are the *NACE Rev. 2* (2-digit level) core business activities included in our dataset are given in the S1 Appendix.

### The network structure of city-activities of globalized firms relations

A central role to our analysis is played by the bipartite network of cities and economic sectors of globalized firms [13]. The scientific literature has several examples of bipartite, or bi-modal networks. Examples include the city-tech knowledge network [20], firm-projects networks [56], predator-prey networks [57], plants-pollinator networks [30] etc. In our analysis we use the data described above to generate an *l* × *k* city-activities matrix **E**, where the matrix element *E*_*c*,*s*_ represents the city *c* that hosts the firms with global presence operating in the economic sector *s* (see next section).

This matrix allows for the construction of an undirected city-activities network by linking each city to the number of firms with presence operating in the different *NACE Rev. 2* (2-digit) sections.

**Fig 1:**
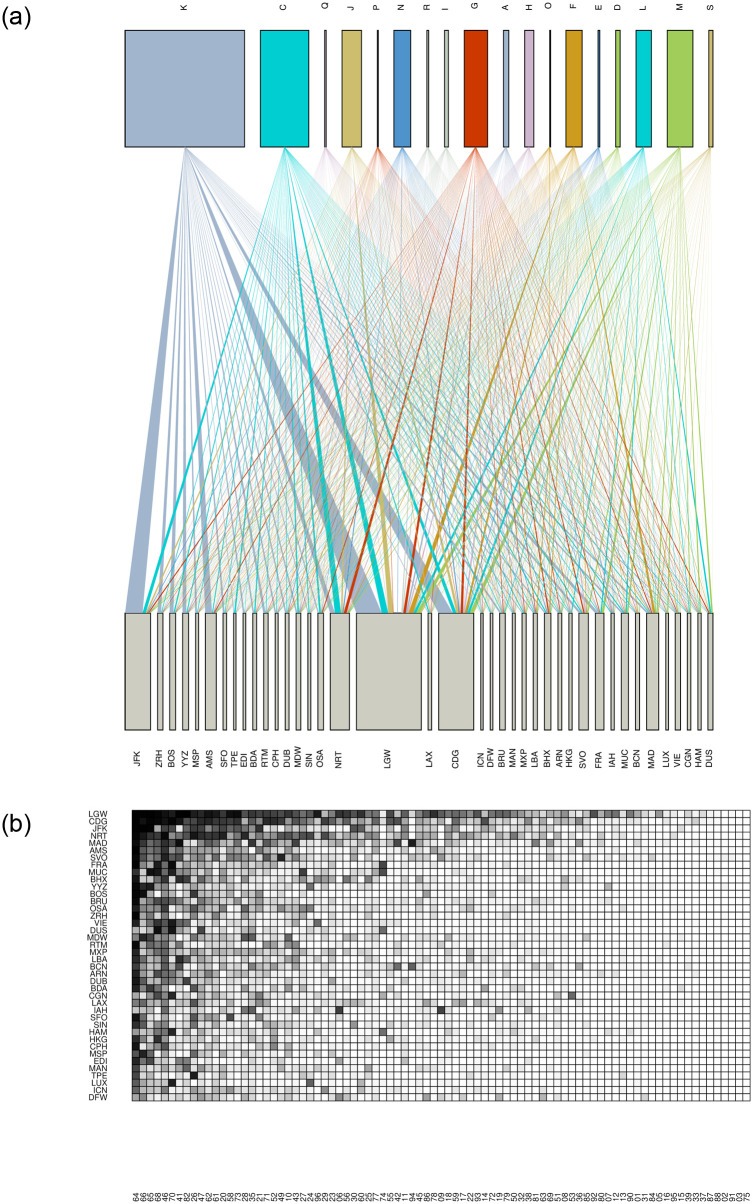
The city-activities of globalized firms network. [a] The bipartite network of cities and economic sections (NACE Rev. 2). [b] The adjacency matrix using the economic activities at NACE Rev. 2 (2-digit). The figures have been generated using R 4.1.2.

In Fig 1 we illustrate the city-activities bipartite network (top) and the adjacency matrix (bottom) for 2010 and for the 40 cities with the highest degree. From this figure, we can easily identify clusters of cities that are linked to specific types of economic activities. The main economic sections of the network are ‘financial and insurance activities’ (K), ‘manufacturing’ (C) and ‘professional, scientific and technical activities’ (M) and they are placed mainly in populous and prosperous cities such as London (IATA code: LGW), Paris (CDG), New York (JFK) and Tokyo (NRT). The adjacency matrix is a matrix representation of the density of the links between a city and its economic activities: darker points indicate cities *c* with higher number of firms operating in sector *s*. The apparent nested/triangular structure [58, 59] highlights the existence of cities that are very well diversified and cities that host only a small set of firms with global presence. Going from the bottom of the matrix to the top, the economic complexity of the cities tends to increase.

## The economic complexity of cities as derived from the economic activities of their globalized firms

To measure the economic complexity of cities based on the diversity and ubiquity of the economic activities of their firms with global presence, we rely on the methodology described in [60], which is based on the *method of reflections* developed by [18].

To quantify the economic complexity of cities, we combine information on the number of globalized firms operating on the different *NACE Rev. 2* sections (2-digit level) and how common these economic activities are across cities.

In short, assuming that we have information for *l* number of cities and *k* economic sectors we fill an *l* × *k* matrix **E**, so that matrix element *E*_*c*,*s*_ is city *c*’s number of firms with global presence (from our dataset of the world’s 3, 000 largest firms and their almost 1 *million* subsidiaries—see section Data on firms with global presence) operating in economic sector *s*. If there are no firms with global presence in sector *s* in city *c*, then *E*_*c*,*s*_ = 0.

From matrix **E** we obtain the *l* × *k* matrix **M**, with matrix elements *M*_*c*,*s*_ = 1 if city *c* has at least one firm with global presence operating in economic sector *s*, and zero otherwise. This matrix can be viewed as the incidence matrix of the bipartite network linking cities to economic activities of firms with global presence (the *city-activities of globalized firms network* discussed in the previous section). From this matrix, following [18], we compute the ECI as a measure that quantifies the structure of city-firms network based on the diversity of cities’ firms with global presence and on the number of cities that have globalized firms operating in a particular economic sector.

To obtain the ECI we sequentially combine the diversification of cities in terms of their economic activities, *k*_*c*,0_ = ∑_*s*_ *M*_*c*,*s*_, and the ubiquity of globalized firms’ economic sectors, *k*_*s*,0_ = ∑_*c*_ *M*_*c*,*s*_, in the following equation over a series of *n* iterations:

$$\begin{array}{c} {\text{ECI}}_{c}=K_{c,n}=\sum_{s}M_{c,s}\frac{1}{\sum_{c}\frac{M_{c,s}}{K_{c,n-2}}} \end{array}$$ (1)

The iteration process eliminates noise and size effects of the ubiquity of globalized firms in relation to which cities they operate and to the diversity of cities that host firms with global presence. We hence provide finer-grained estimates of the complexity of cities using information on the economic activities of firms with global presence operating in these cities. The iteration process converges when the ranking of cities and economic sectors does not change from one iteration to another i.e. when the information of the city-activities of globalized firms network is fully captured by the index. In a Markov-chain setting, the iterative method described above is an approximation of a fixed-point theorem. Using linear algebra analysis the ECI could be computed for each city as the second eigenvector of the square matrix **MM**^*T*^ (see also [20]; for the computations we have used R 4.1.2).

**Fig 2:**
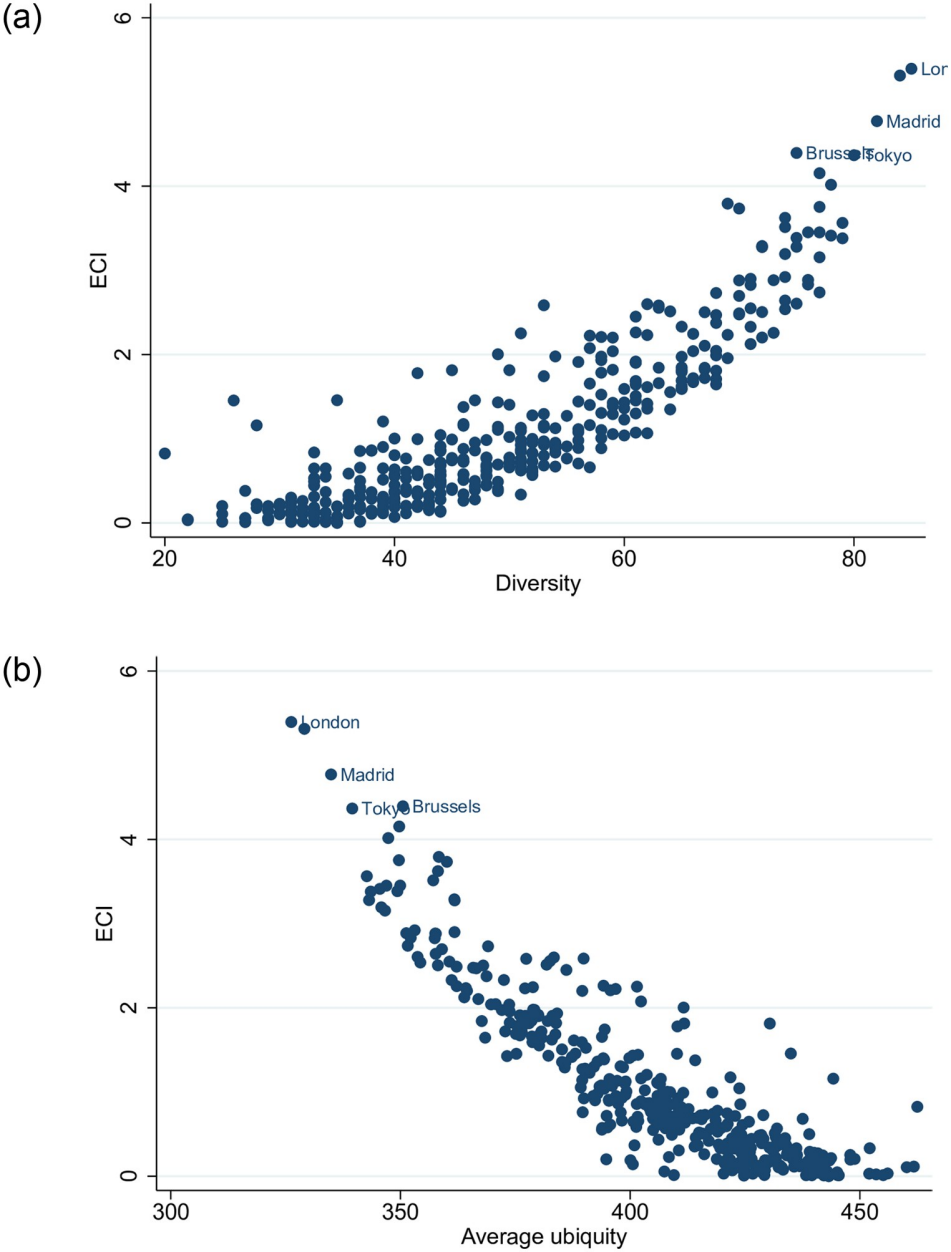
Cities’ economic complexity (ECI), diversity and average ubiquity. [a] ECI vs Diversity; [b] ECI vs Average ubiquity. The figures have been generated using StataSE 14.

The ECI reflects the composition of a city’s “pool” of firms (economic activities) with global presence, taking into account the composition of the “pools” of all other cities. Cities with low diversity that host economic activities that are being hosted by many other cities have relatively low economic complexity scores. More complex cities host non-ubiquitous economic sectors and exhibit higher diversity (Fig 2).

## The economic complexity of cities across the world

**Fig 3:**
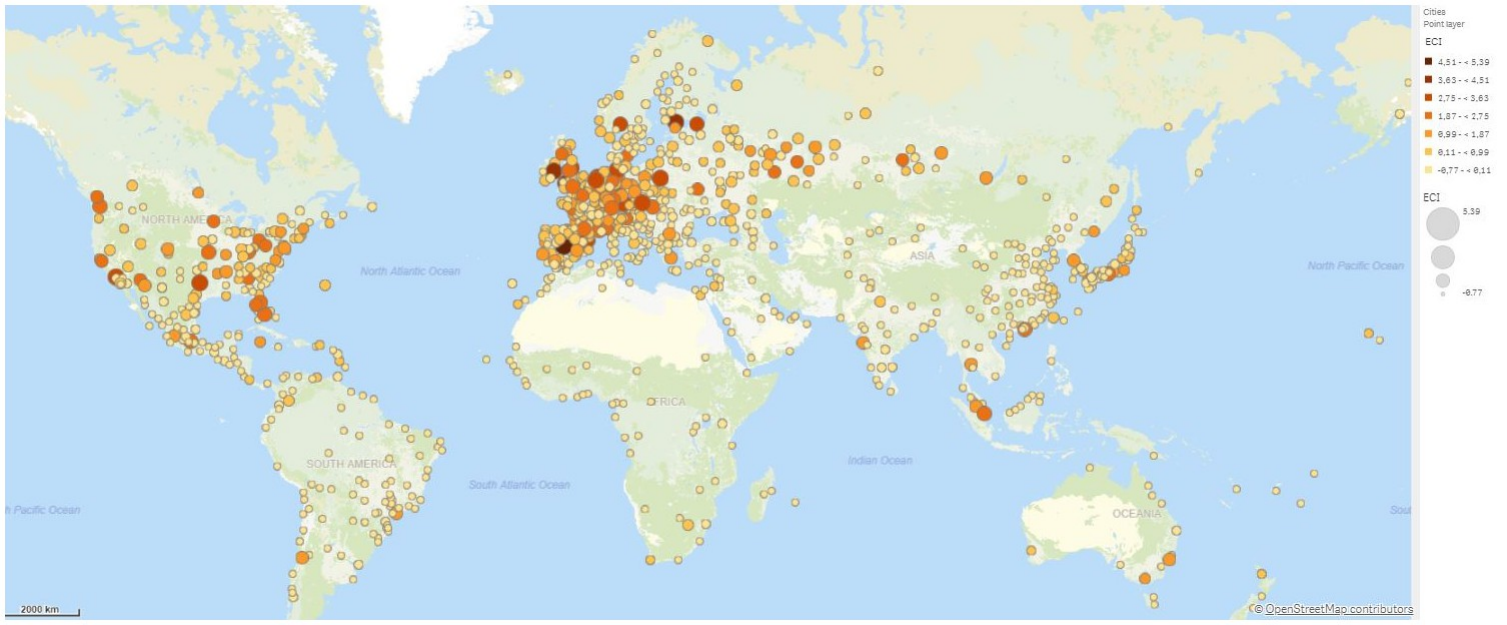
Cities economic complexity index (ECI) across the largest cities. Cities depicted in dark orange and big circle have a high ECI value (Data for 2010). The figure has been generated using Qlik Sense. ©OpenSTreetMap contributors.

**Fig 4:**
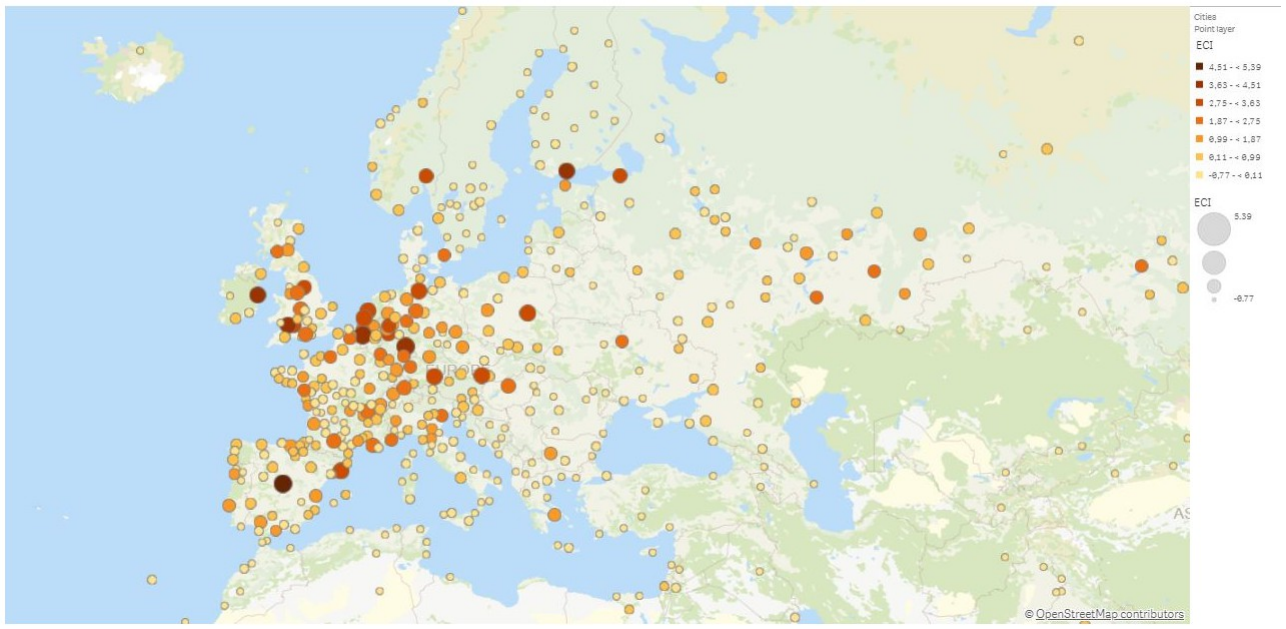
Cities economic complexity index (ECI) in Europe. Cities depicted in dark orange and big circle have a high ECI value (Data for 2010). The figure has been generated using Qlik Sense. ©OpenSTreetMap contributors.

In Figs 3 and 4 we map the ECI across cities globally and in Europe respectively. We see rather clearly that complexity is unevenly distributed in the world and that the most complex cities in terms of economic activities seem to be located in Europe and North America. In contrast, most cities in Africa, South America and Asia have much lower ECI. Table 1 lists the five cities with the highest ECI scores.

**Table 1:** List of the top five cities with the highest ECI values.

City	Country	ECI
London	Great Britain	5.394
Paris	France	5.314
Madrid	Spain	4.772
Brussels	Belgium	4.395
Tokyo	Japan	4.367

ECI: Economic Complexity Index; Data for 2010.

**Fig 5:**
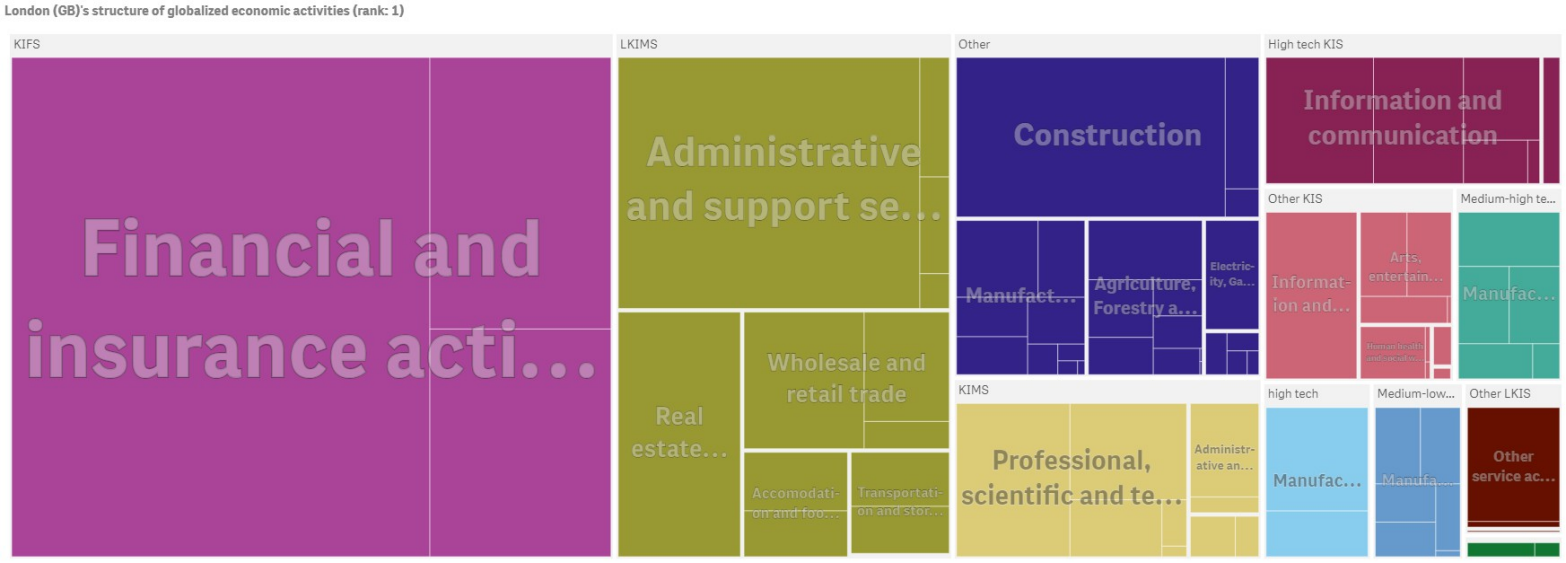
The economic activities of London (GB)’s firms with global presence. Paris ranks second in the economic complexity index of cities in 2010 (ECI: 5.394). The figure has been generated using Qlik Sense.

**Fig 6:**
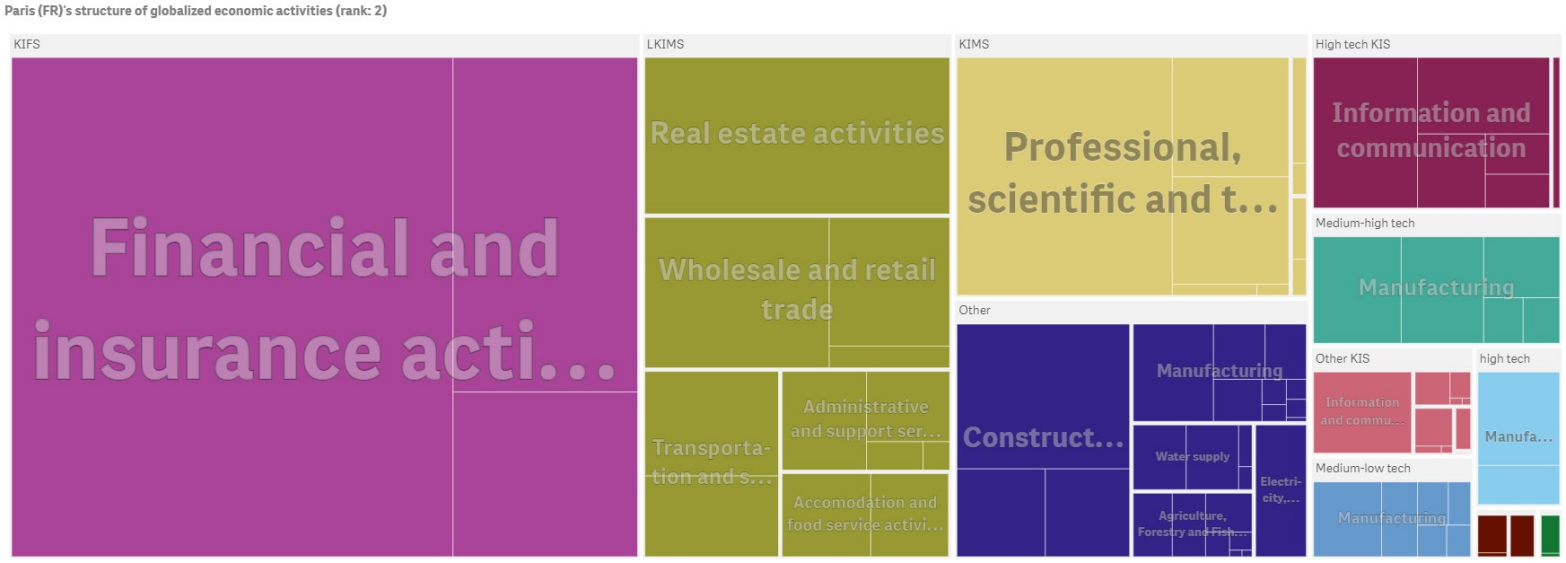
The economic activities of Paris (FR)’s firms with global presence. Paris ranks second in the economic complexity index of cities in 2010 (ECI: 5.314). The figure has been generated using Qlik Sense.

London and Paris are the two countries with the highest diversification of firms with global presence in non-ubiquitus *NACE Rev. 2* (2-digit) economic activities. Figs 5 and 6 show that both cities host globalized firms with highly diversified economic activities ranging from the Knowledge-Intensive (KIFS) ‘*Financial and insurance activities*’ to Knowledge-Intensive Market Services (KIMS) such as *NACE Rev. 2* (2-digit) division 73 ‘*Advertising and market research*’, division 71 ‘*Architectural and engineering activities; technical testing and analysis*’ and to high-tech manufacturing division 26 ‘*Manufacture of computer, electronic and optical products*’ and division 21 ‘*Manufacture of basic pharmaceuticals products and pharmaceutical preparations*’ (See Eurostat indicators on high-tech industry and knowledge-intensive services by NACE Rev. 2 (https://ec.europa.eu/eurostat/cache/metadata/Annexes/htec_esms_an3.pdf)). Both cities also host globalized firms operating in high-tech Knowledge-Intensive Services (KIS) such as *NACE Rev. 2* (2-digit) division 62 ‘*Computer programming, consultancy and related activities*’ and division 61 ‘*Telecommunications*’.

**Fig 7:**
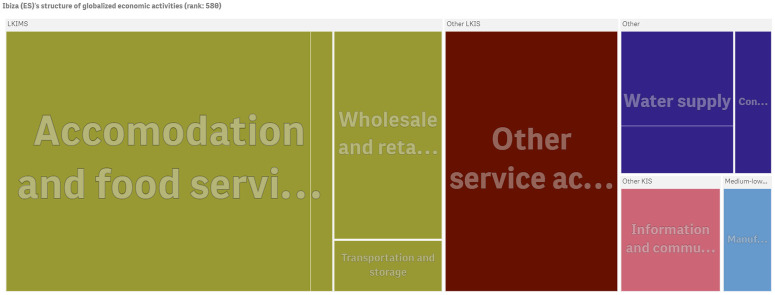
The economic activities of Ibiza (ES)’s firms with global presence. Ibiza ranks 580th in the economic complexity index of cities in 2010 (ECI: -0.407). The figure has been generated using Qlik Sense.

**Fig 8:**
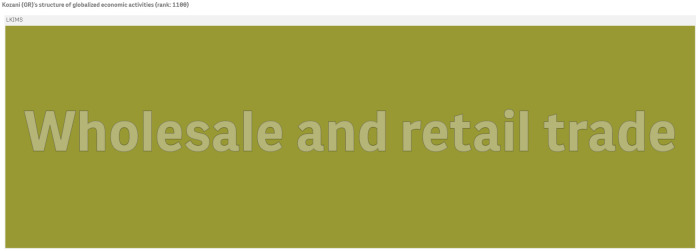
The economic activities of Kozani (GR)’s firms with global presence. Kozani ranks 1100th in the economic complexity index of cities in 2010 (ECI: -0.763). The figure has been generated using Qlik Sense.

For conceptualizing further the ECI for cities we depict the structure of economic activities of globalized firms operating in Ibiza and Kozani (a small city in Greece) in Figs 7 and 8 respectively. Ibiza ranks 580th out of 1170 cities and Kozani appears in the 1100th position. Ibiza is less diversified than London and Paris and tends to focus on tourism and retail industries that are considered less knowledge intensive market services (LKIMS).

At the end of the ECI spectrum, Kozani (GR) hosts only one firm with global presence that operates in the ubiquitous *NACE Rev. 2* (2-digit) LKIMS activity 47 ‘*Retail trade, except of motor vehicles and motorcycles*’. This economic activity appears in 654 other cities and it ranks 3rd in ubiquity (the most ubiquitous economic activity is the LKIMS *NACE Rev. 2* (2-digit) division 46 ‘*Wholesale trade, except of motor vehicles and motorcycles*’).

## Linking economic complexity to socio-economic indicators of cities

In this section we explore whether the proposed index captures various determinant factors of cities’ competitiveness in attracting FDI. More specifically, we link our index to population, Gross Domestic Product (GDP) and total employment capturing cities’ size and level of development, education and patent applications as a proxy for human capital and innovation, and internet and transport infrastructure. In doing so, we simply present correlations of our index with each of the above variables, thus we do not make any arguments as to causation. We just aim at illustrating that the ECI for cities is useful in covering various aspects of urban competitiveness in attracting globalized firms. In the following section, we undertake a more formal analysis to show the interplay between the ECI and measures of cities’ resilience.

We employ data for cities from the European Commission (EC)’s *Urban Data Platform Plus* and two samples of EU NUTS 3 regions (2010 classification) for which ESPON (European Spatial Planning Observation Network) and Eurostat have systematic data (The ESPON EGTC is a European Grouping on Territorial Cooperation and since 2002 is building a pan-European knowledge base related to territorial dynamics –see https://www.espon.eu/. It is database portal that ensures the availability of harmonised and accurate data on the European territory and neighbouring countries).

### Complex economic activities of globalized firms concentrate in large and prosperous cities

**Fig 9:**
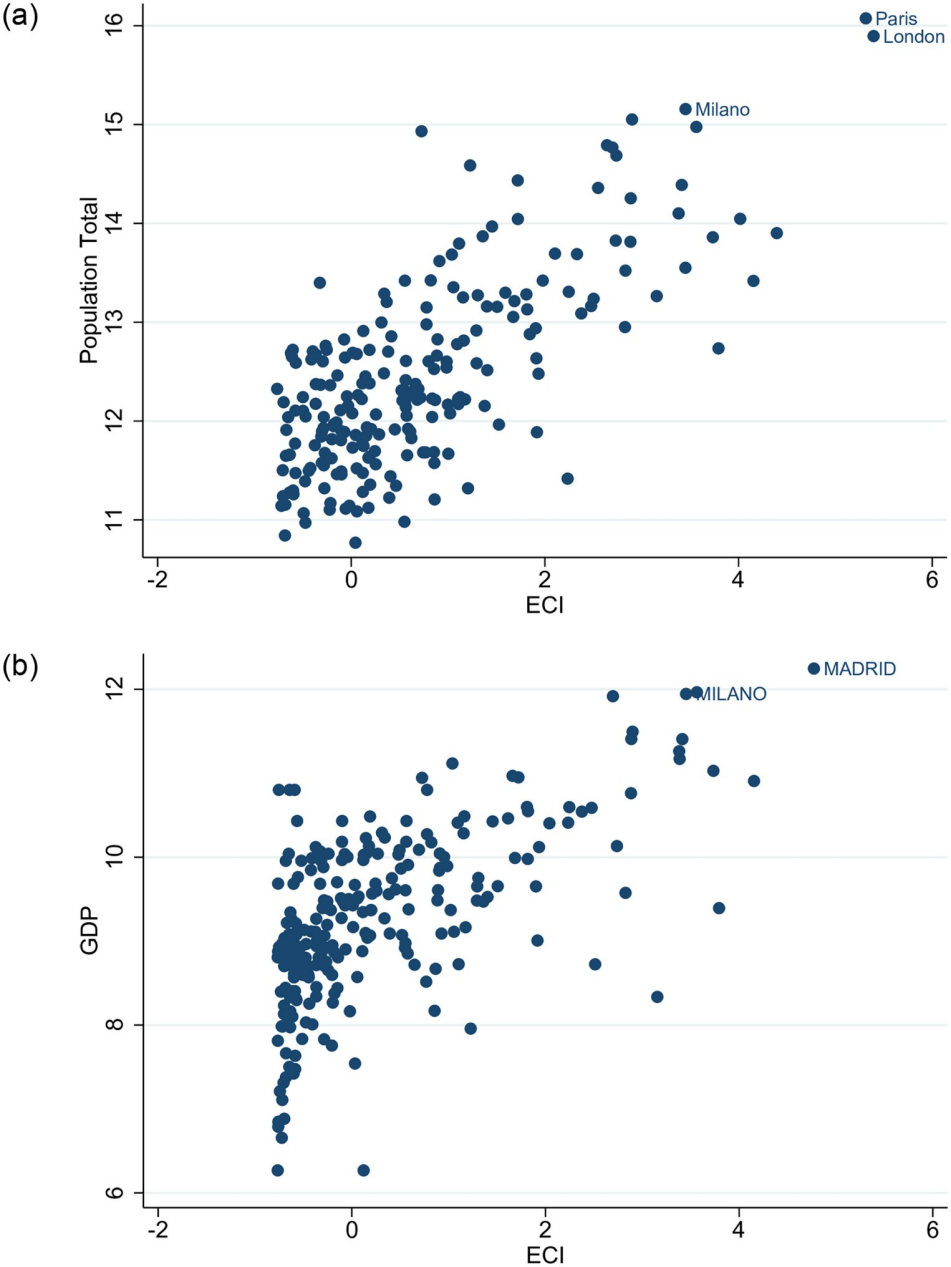
Cities economic complexity (ECI), population and development. [a] ECI vs Population; [b] ECI vs GDP. The figures have been generated using StataSE 14.

Fig 9 depicts the strong and positive correlations between the economic complexity of cities and their population [the Pearson correlation coefficient for ECI and total population is 0.735 (p-value <1%); the Spearman coefficient is 0.628 (p-value <1%)] and level of development proxied by GDP [Pearson: 0.622 (p-value <1%); Spearman: 0.613 (p-value <1%)]. We consider these two measures as broader proxies of economic development. Complex cities are rich cities and as a result they attract foreign capital, firms and people. The literature on the locational determinants of FDI throws light on the important role of economies of scale and agglomeration effects in firms’ FDI decisions [61, 62]. Cities with a large market and high level of economic development, which in turn imply good economic institutions, play a crucial mediating role in attracting FDI [63].

### Complex economic activities of globalized firms concentrate in cities with increasing concentration of jobs, human capital and innovation

**Fig 10:**
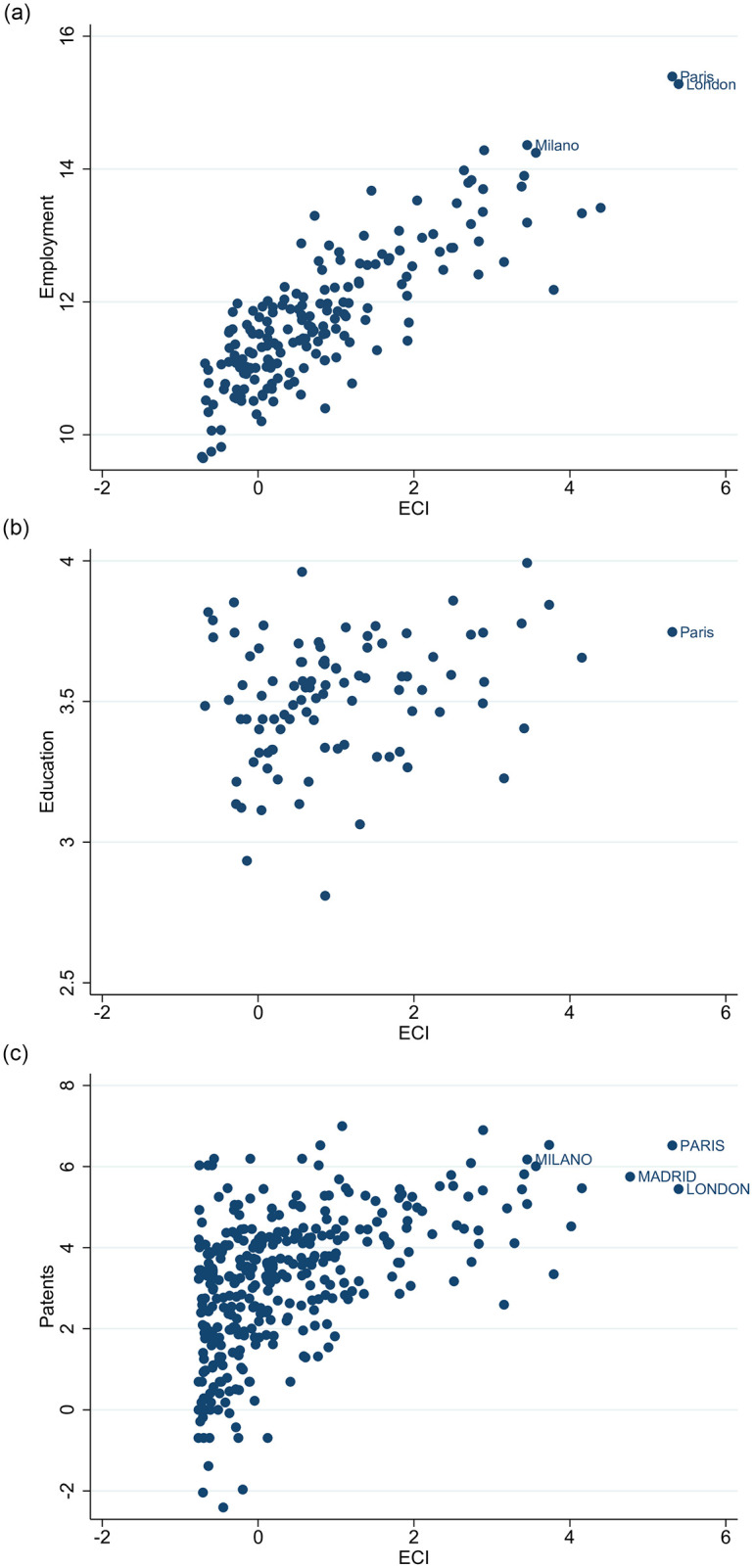
Cities economic complexity (ECI), employment, education and innovation. [a] ECI vs Total employment; [b] ECI vs Tertiary education; [c] ECI vs Patents. The figures have been generated using StataSE 14.

Fig 10 depicts the positive correlations between the economic complexity of cities and their level of employment [Pearson: 0.842 (p-value <1%); Spearman: 0.789 (p-value <1%)], human capital [Pearson: 0.262 (p-value <1%); Spearman: 0.234 (p-value <1%)] and innovation [Pearson: 0.510 (p-value <1%); Spearman: 0.524 (p-value <1%)] proxied by the number of patent applications to the European Patent Office (EPO). Complex cities give incentives and opportunities to skilled people and the combination of a skilled labor force as well as the availability of such jobs can more easily lead to innovation.

Hidalgo [17] argues that what Kuznets called ‘measure of our ignorance’ in his Nobel Prize acceptance speech is actually our individual mental capacity, our ‘personbytes’. The ECI developed with data on the export basket of countries captures the ‘personbytes’ of skill/knowledge to produce complex products i.e. products that only a few highly developed economies make. Hence, more ‘personbytes’ lead to more innovation which in turn can be measured through the economy’s level of economic complexity giving us insight about the economy’s capacity to generate innovation and its ability to apply those innovations in developing new products [64]. Here, we adapt the economic complexity methodology to the network of cities and economic activities of their globalized firms introducing a similar reasoning about how the ECI of cities proxies for the level of human capital, infrastructural capital, institutions (proxied by the level of development), knowledge and innovation capacity of their economies, as reflected in the structure of economic activity. More specifically, we translate ‘personbytes’ into the capabilities (the various determinants of urban competitiveness for economic gain) and policy interventions to attract globalized firms and investors, and preparedness to face negative economic shocks. As such, the index captures more elaborate aspects of a city’s economic activity compared to GDP per capita, which is a broader measure of prosperity capturing several economic, social, cultural and institutional aspects of a city.

### Complex economic activities of globalized firms concentrate in cities with robust technology and transport infrastructure

Besides high quality institutions, ‘physical’ factors such as transport and communication infrastructure also underpin local business operating conditions and determine the locational decisions of firms for FDI. “*Good infrastracture is a necessary condition for foreign investors to operate successfully, regardless of the type of FDI*.” [63, p. 10]. Then, the economies of scale and agglomeration effects appear also here: “*Competitive cities tend to attract a disproportionate share of total financing for infrastructure, driven by larger local equity pools, greater perceived creditworthiness, and access to a larger range of financing sources due to scale (e.g., large cities can tap the bond market).”* [16, p. 11]

**Fig 11:**
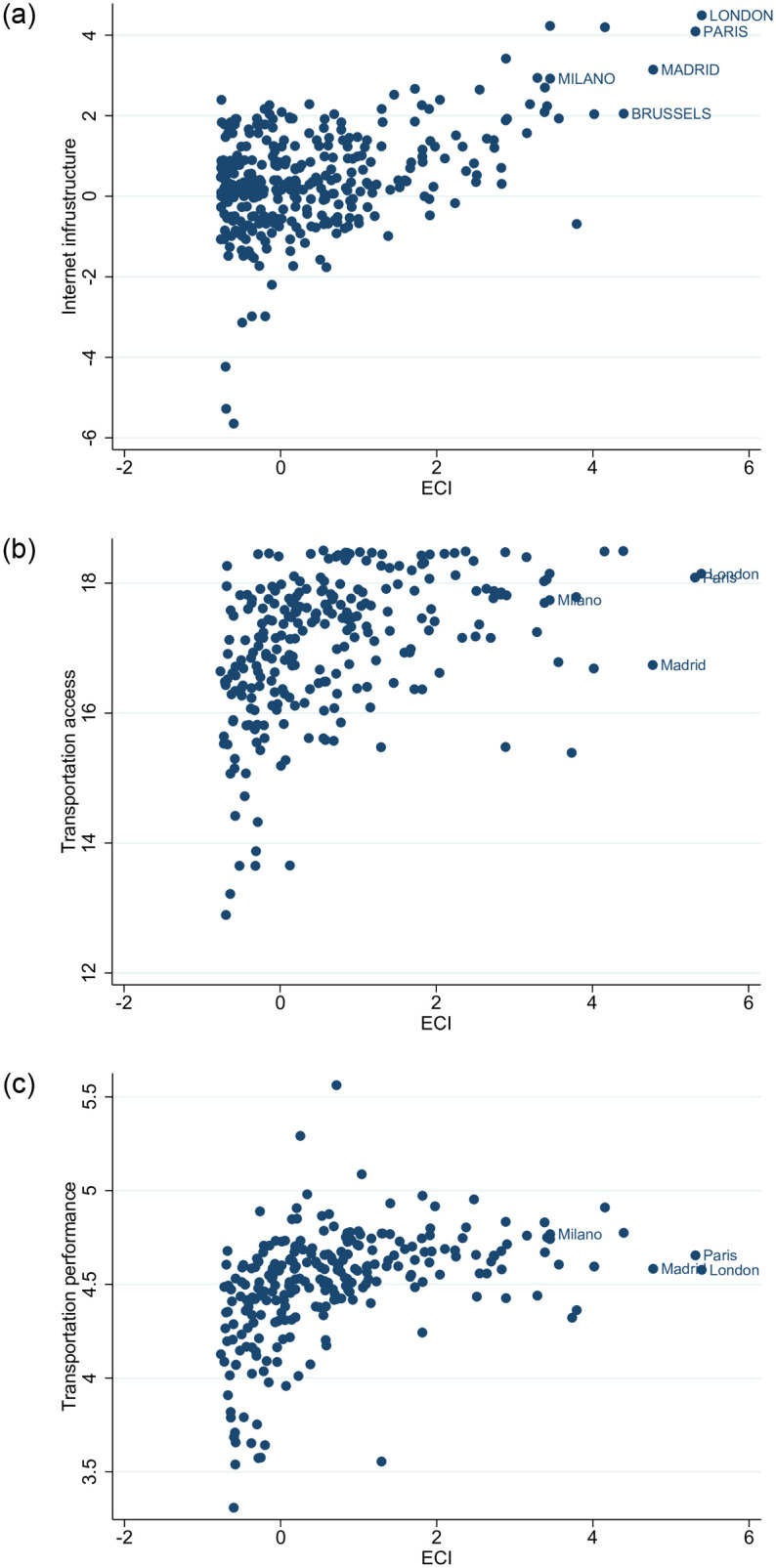
Cities economic complexity (ECI), digital and transport infrastructures. [a] ECI vs Internet infrastructure; [b] ECI vs Transportation accessibility; [c] ECI vs Transportation performance. The figures have been generated using StataSE 14.

Fig 11 depicts the correlations between the economic complexity of cities and their Internet infrastructure [Pearson: 0.445 (p-value <1%); Spearman: 0.275 (p-value <1%)], transportation access [Pearson: 0.397 (p-value <1%); Spearman: 0.481 (p-value <1%)] and transportation infrastructure [Pearson: 0.416 (p-value <1%); Spearman: 0.556 (p-value <1%)]. As was the case with the rest of the variables, there is again a positive correlation.

Internet infrastructure index is from ESPON and according to the source, it is a composite indicator on the Internet infrastructure, calculated as the average of the following Internet infrastructure indicators: international Internet backbone capacity, peak traffic at IXPs and IP addresses all at regional level. Data for the transportation accessibility and performance variables have been drawn from the European Commission’s *Urban Data Platform Plus*. The two indicators are produced by the LUISA Modelling Platform [65]. Transportation access refers to daily accessibility and indicates the amount of people that live within four hours of driving from the location at hand. Transportation performance (Transport Performance indicator) measures the ratio between the number of people that can be reached within a 90-minute drive (accessible population) versus the number of people living within a radius of 120 km (nearby population or proximity) from a specific location.

All the above measures captured by the ECI reflect different types of cities’ prosperity and indicate how cities can become competitive in attracting complex economic activities by developing the necessary infrastructure that further reinforces their level of economic complexity thus entering a virtuous cycle of development.

## Complex economic activities of globalized firms condition the resilience of cities

In this section, we focus in one particular relationship between the ECI for cities and a measure that is essential for cities, i.e., their resilience. Unlike the previous section, in this section we conduct a regression analysis in order to be able to confer the correlation between our index and resilience of cities to negative economic shocks.

### Econometric specification

To analyze the effect of complex economic activities of globalized firms to resilience of cities we apply a linear regression model with fixed effects at NUTS 2 level (we use StataSE 14). This allows us to exploit within NUTS 2 variation and thus to account for several unobservables that could confound our estimations. Following the relevant literature we measure regional economic resilience by employment [38, 52, 66–68]. We use the data for EU NUTS 3 regions from Eurostat (2016 classification) and we check the robustness of our findings with the ESPON database (We use the European Commission’s ‘NUTS converter’ tool, available here https://urban.jrc.ec.europa.eu/nutsconverter/#/, to convert ECI data to 2016 NUTS 3 version).

Given the availability of controls, the sample for the baseline specification covers 365 NUTS 3 regions (2016 classification). For the estimation we use robust standard errors clustered at the NUTS 3 level. We construct our index of employment resilience using two years, i.e., the years 2006 and 2012. The index reflects the ability of a region to return to prior employment levels before the emergence of a shock. The reason for choosing this range of years is so that we want to exploit the emergence of the Global Financial Crisis, emerging during 2007–2008, which climaxed with the collapse of the Lehman Brothers. This was a major shock, the strongest recession after the Great Depression, which caused major shifts in the production structure of countries, cities and all economic units all over the world.

**Fig 12:**
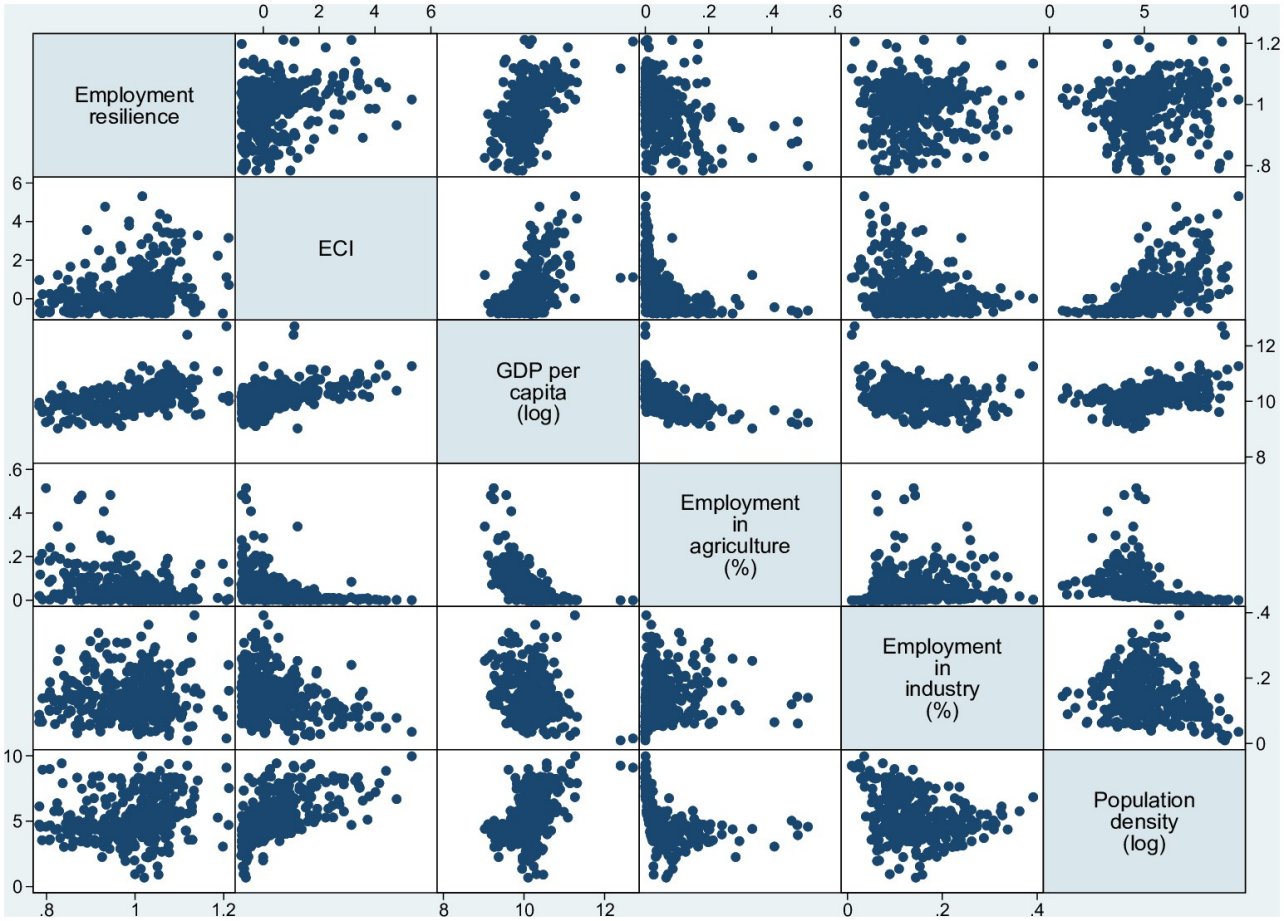
Correlation matrix. The figure has been generated using StataSE 14.

We regress the baseline specification described by the following equation:

$$\begin{array}{c} \frac{Emp_{i,2012}}{Emp_{i,2006}}=a_{0}+\beta_{1}ECI_{i}+\beta_{k}controls_{i}+u_{i} \end{array}$$ (2)

where the dependent variable $\frac{Emp_{i,2012}}{Emp_{i,2006}}$ is the ratio of employment in 2012 divided by employment in 2006 and captures the regional resilience as the change in employment from 2006 to 2012. The employment resilience of city *i* depends on the city’s economic complexity (*ECI*_*i*_) and a set of city-level controls, i.e., (log) Gross Domestic Product per capita (measured in PPS and denoted as GDP per capita) to capture the level of region/cities’ development; the share of employment in agriculture and the share of employment in industry to capture differences in the structure of the regional economies; the (log) population density to capture size effects; the urban-rural typology including remoteness (The urban-rural typology classifies all NUTS 3 regions into the following five categories: 1. predominantly urban regions (value: 1); 2. intermediate regions, close to a city (value: 21); 3. intermediate, remote regions (value: 22); 4. predominantly rural regions, close to a city (value: 31); 5. predominantly rural, remote regions (value: 32). For methodological details see https://ec.europa.eu/eurostat/statistics-explained/index.php?title=Archive:Regional_typologies_overview&oldid=68944#Urban-rural_typology_including_remoteness); the dummy variable ‘metropolitan regions’ that covers all NUTS 3 regions with at least 250, 000 inhabitants (https://ec.europa.eu/eurostat/web/metropolitan-regions/background). *u*_*i*_ is the stochastic term. Fig 12 depicts the correlation matrix and Table 2 shows the summary statistics of our key variables.

**Table 2:** Summary statistics.

	Obs.	Mean	Std. dev.	Min	Max
Employment Resilience	365	0.992	0.081	0.784	1.212
ECI	365	0.371	1.094	-0.782	5.314
(log) GDP per capita	365	10.12	0.419	9.029	12.70
Employment in agriculture (%)	365	0.056	0.074	0.000	0.514
Employment in industry (%)	365	0.143	0.066	0.009	0.392
(log) Population density	365	5.227	1.668	0.693	9.967
Urban-rural typology	365	16.86	12.18	1	32
Metropolitan regions	365	0.553	0.498	0	1

### Econometric results

Our benchmark analysis is conducted in Table 3. In Column (1) we present the unconditional correlation between the ECI for cities and our measure of resilience for the sample of 365 cities. In Column (2) we control for GDP per capita. Column (3) extends the analysis by accounting for the shares of employment in agriculture and industry, while in Column (4) we control for population density. Columns (5) and (6) include the ‘urban-rural typology’ and ‘metropolitan regions’ respectively to control for the impact of predominantly urban areas and big cities such as capital cities (Our set of controls is limited as we do not plan to undertake a fully-fledged econometric analysis, but mostly to illustrate the usefulness of the index. We thus choose controls that are directly related to employment resilience). The results in all columns are strong and statistically significant. Moreover, the coefficient and the standard errors remain stable across all specifications. The coefficient in Column (6), which we will henceforth report as the benchmark regression, since it contains the full set of controls, suggests that the expected increase of the ratio of employment in 2012 over 2006 is 0.01 (mean: 0.992; standard deviation: 0.081) when the ECI increases by 1 unit (mean: 0.371; standard deviation: 1.094), thus suggesting a non-trivial pace of faster recovery for complex cities.

**Table 3:** Regression results.

Dependent variable: Employment resilience	(1)	(2)	(3)	(4)	(5)	(6)
ECI	0.012***	0.005*	0.008**	0.010**	0.011***	0.010**
	(0.003)	(0.003)	(0.003)	(0.004)	(0.004)	(0.004)
GDP per capita (in logs)		0.076***	0.082***	0.085***	0.087***	0.084***
		(0.011)	(0.011)	(0.011)	(0.011)	(0.011)
Employment in agriculture (%)			0.101	0.028	0.010	0.047
			(0.068)	(0.104)	(0.108)	(0.108)
Employment in industry (%)			0.075	0.046	0.045	0.082
			(0.058)	(0.059)	(0.060)	(0.066)
Population density (in logs)				-0.008	-0.006	-0.007
				(0.006)	(0.007)	(0.007)
Urban-rural typology					0.000	0.001
					(0.001)	(0.001)
Metropolitan regions						0.019**
						(0.009)
Observations	365	365	365	365	365	365
F-stat	147.0	64.55	220,128	994.7	65.33	84.76
R-squared	0.844	0.865	0.866	0.867	0.867	0.870

Ordinary least squares regression with fixed effects (at NUTS 2 level) and robust standard errors clustered on the NUTS 3 level (in parentheses).

* p<0.10,

** p<0.05,

*** p<0.01

**Fig 13:**
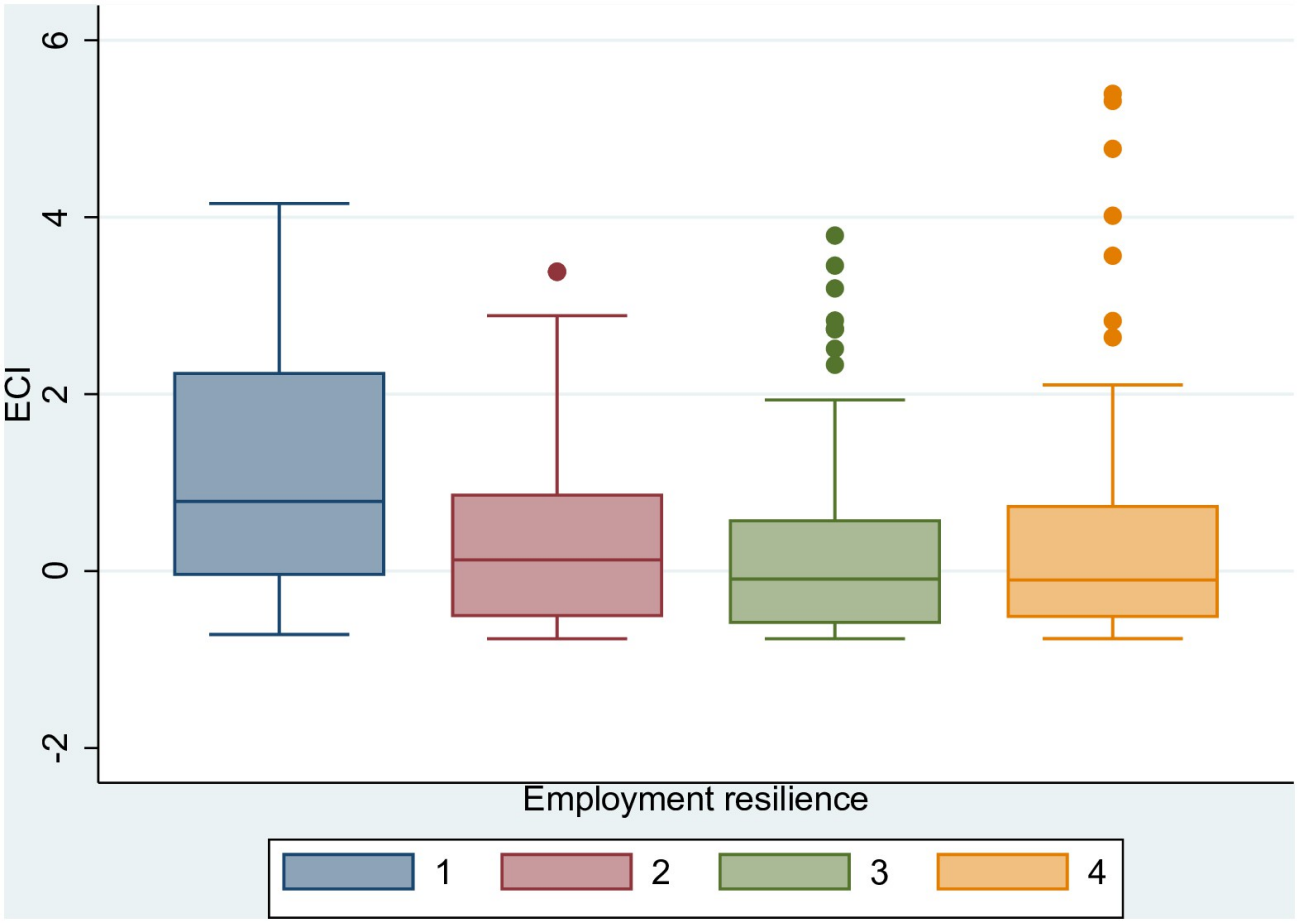
Employment resilience and ECI. Employment resilience measured using the index developed by ESPON [69]: 1 = resistant to the economic crisis; 2 = recovered from the economic crisis; 3 = not recovered but experienced economic upturn; 4 = not recovered and still experiencing economic downturn. The figure has been generated using StataSE 14.

**Fig 14:**
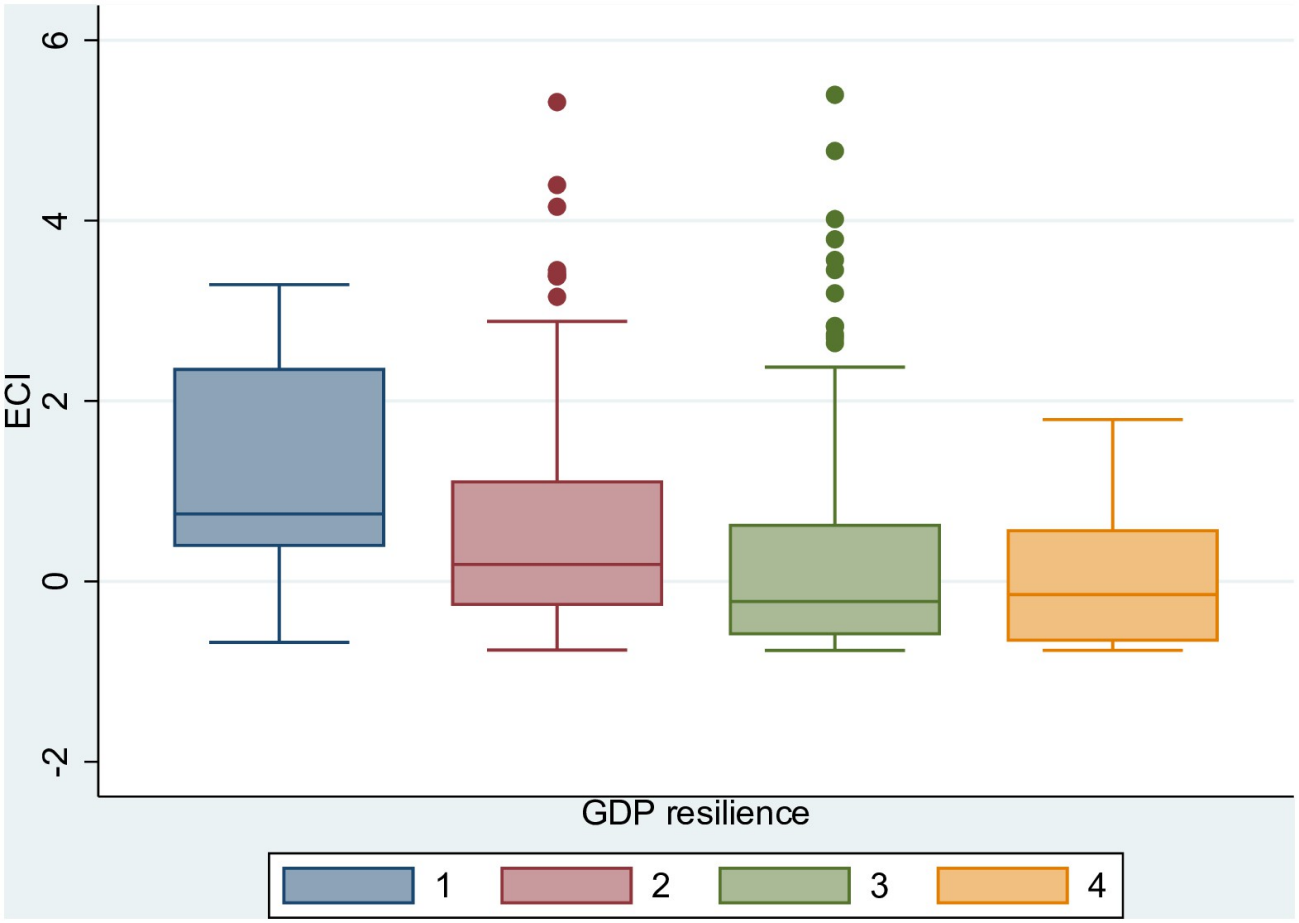
GDP resilience and ECI. GDP resilience measured using the index developed by ESPON [69]: 1 = resistant to the economic crisis; 2 = recovered from the economic crisis; 3 = not recovered but experienced economic upturn; 4 = not recovered and still experiencing economic downturn. The figure has been generated using StataSE 14.

As a robustness check of the above analysis and to further support our finding that complex cities have the ability to recover relatively quickly from an adverse shock, we use Fig 13 to show the negative relationship of ECI with the four categories of the ESPON employment resilience index (i.e. a positive relationship of ECI with employment resilience). The resilience index takes discrete values from 1 (resistant to the economic crisis) to 4 (not recovered and still experiencing economic downturn) [69]. The estimated coefficient of ECI adopting an ordered logit regression model is −0.536 with p-value <0.01 (controlling for GDP in logs and using robust standard errors clustered at NUTS 3 level). According to the methodology of ESPON [69], a resilient region is considered to be one that has either resisted the economic crisis, in that it has not experienced a decline in economic activity, or has recovered from a downturn following the economic shock to regain pre-crisis levels of activity. The economic activity is primarily measured through the indicator of number of persons employed (employment resilience), but ESPON also includes the GDP indicator (GDP resilience) as a comparator (Section 2 in ESPON’s scientific report describes the methodological approach taken in measuring the economic resilience of regions [69]). Fig 14 shows again a positive relationship between the economic complexity of cities and their ability to resist negative economic shocks using the GDP indicator of resilience this time. The respective ordered logit regression model gives an estimated coefficient of −0.314 for the ECI (p-value <0.01 and robust standard errors clustered at NUTS 3 level).

## The mechanics of the ECI

Overall our results suggest that ECI is positively correlated with employment resilience. To understand the mechanics behind this relationship we turn our focus to the two main elements reflected in the ECI, i.e., the diversity and non-ubiquity of activities, are the drivers behind the ability to recover faster.

Unlike GDP, which is a broader proxy of economic prosperity, ECI is capturing the diversification in the range of activities. Considering an example with two countries, e.g., a service and infrastructure-based economy (country A) and a resource-rich economy (country B), they can both potentially experience similar levels of income per capita. However, the former (country A) would be much less vulnerable to an exogenous shock compared to the latter example (country B). The reason is that diversification in the production, captured by a high ECI and not necessarily reflected in a high GDP, can act as the safety net and can mitigate the adverse effects of a recession via diversification and via mitigating the shocks on employment. In light of the recent shock of COVID-19, we observe that whereas it generated difficulties in the supply chain and adversely affected the production process, at the same time it gave rise to a whole new range of online services and thriving sectors. Therefore, countries more diversified across various sectors could compensate losses in one sector via directing resources and factors of production to other sectors. Moreover, the ubiquity part of the index (i.e., the fact that low ubiquity leads to a high ECI) suggests that in the face of a shock and in the rise of new emerging sectors, cities with high ECI can grasp most of the rents associated with those activities and generate many employment opportunities. Overall, having a regional economy that is sufficiently diversified, a shock will have little macroeconomic effect [70].

## Conclusions

Based on the economic complexity methodology we compute the ECI for cities reflecting the diversity and ubiquity of cities’ firms (economic activities) with global presence [18, 71]. We illustrate the value added of our index by correlating it to a set of EU cities’ socio-economic indicators and regressing it on the ratio of total employment in 2012 over total employment in 2006 capturing in this way the employment resilience of cities against the economic crisis of 2008. There are four main results: (*i*) economic complexity of cities is a good proxy for the scale of a city’s economy as measured by its population and GDP; (*ii*) to better refine point (i), the ECI for cities is positively correlated with measures such as total employment, the share of tertiary education, number of patent applications, internet infrastructure, transportation access and performance; and (*iii*) the ECI illustrates that complex economic activities of globalized firms condition the resilience of cities and that complex cities tend to be the ones that either resisted the economic crisis or regained quickly their pre-crisis levels of employment and GDP.

All the above results and especially the latter, uncover important policy implications as they suggest that diversity and non-ubiquity of globalized firms’ economic activities is the way to secure resilience of a city, an action that can be undertaken and achieved in a city of any scale. Cities’ characteristics is undoubtedly of great importance when firms make their location decisions about their FDI. Local governments’ policies should embrace diversity and avoid focusing their urban planning on a particular economic sector. Cities should have an economic development policy that targets complex economic sectors through improving key influential factors of FDI such as access to markets, efficient regulations and procedures, inclusive institutions, high-quality infrastructure and well-educated labour force.

However, our findings come with limitations. A first one is that national and local firms are not considered in the analysis and only data on multinational firms and their subsidiaries are explored. An additional limitation regards the sample of firms considered that is restricted to the world’s 3,000 largest multinational firms. Given that big multinational firms tend to choose larger cities for their subsidiaries results in considering the 1,169 largest cities in our sample (of the 1,860 cities with at least 300,000 inhabitants in 2018 included in World Urbanization Prospects (WUP) [72]). The above imply that our results may not be applicable when considering all firms in all urban areas and regions of the world.

In future research a broader sample of firms and cities can be analyzed including smaller multinationals and local firms operating in smaller cities worldwide. An additional venue for future research could be the application of the proposed methodology on more granular data for the economic activities (e.g., 3-digit level of *NACE Rev. 2*).

## Supporting information

S1 Appendix Classification of firms with global presence. (PDF)
