# Supplementary material for: Economic complexity of cities and its role for resilience
Source: PLoS One. 2022 Aug 4;17(8):e0269797. doi: 10.1371/journal.pone.0269797 (PMC9352037; doi:10.1371/journal.pone.0269797)
Supplement: S1 Appendix — (PDF) [file pone.0269797.s001.pdf]

**S1 Appendix. Classification of firms with global presence.** Firms are classified following the *NACE Rev. 2* statistical classification of economic activities in European Community (2-digit level) based on their core business activity. Our dataset includes the following sections: A – '*Agriculture, forestry and fishing*', B – '*Mining and quarrying*', C – '*Manufacturing*', D – '*Electricity, gas, steam and air-conditioning supply*', E – '*Water supply: sewerage, waste management and remediation activities*', F – '*Construction*', G – '*Wholesale and retail trade; repair of motor vehicles and motorcycles*', H – '*Transportation and storage*', I – '*Accommodation and food service activities*', J – '*Information and communication*', K – '*Financial and insurance activities*', L – '*Real estate activities*', M – '*Professional, scientific and technical activities*', N – '*Administrative and support service activities*', O – '*Public administration and defence; compulsory social security*', Q – '*Human health and social work activities*', R – '*Arts, entertainment and recreation*', S – '*Other service activities*'.
